# A rare cause of persistent hyperparathyroidism

**DOI:** 10.1002/ccr3.3568

**Published:** 2020-11-20

**Authors:** Laura Iconaru, Linda Spinato, Ruth Duttmann, Anne‐Sophie Hambye, Arnaud Devriendt, Maxime Herchuelz, Celine Mathey, Rafik Karmali, Pierre Bergmann

**Affiliations:** ^1^ Department of Endocrinology CHU Brugmann Université Libre de Bruxelles Brussels Belgium; ^2^ Department of Otorhinolaryngology CHU Brugmann Université Libre de Bruxelles Brussels Belgium; ^3^ Department of Anatomopathology CHU Brugmann Université Libre de Bruxelles Brussels Belgium; ^4^ Department of Nuclear Medicine CHU Brugmann Université Libre de Bruxelles Brussels Belgium; ^5^ Department of Radiology CHU B←rugmann Université Libre de Bruxelles Brussels Belgium; ^6^ Department of Nuclear Medicine CUB Erasme Université Libre de Bruxelles Brussels Belgium

**Keywords:** choline PET/CT, ectopic adenoma, hyperparathyroidism

## Abstract

In a case of patient with persistent hypercalcemia after parathyroidectomy, different imaging techniques and particularly 18F‐fluorocholine PET/CT are important to localize the adenoma even in a very unusual location.

## Graphical abstract

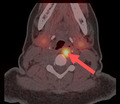

## 1 INTRODUCTION

We present a case with a persistent hypercalcemia after near total cervical parathyroidectomy. A technetium (^99m^Tc) sestamibi scan was performed, but the result was not conclusive. The patient underwent a choline PET/CT, which showed a left retropharyngeal active nodule corresponding to a possible ectopic parathyroid tissue. The patient had a successful transoral removal of the lesion with normalization of serum calcium and iPTH after surgery.

In conclusion, we report a case of ectopic retropharyngeal parathyroid adenoma, for which the 18F‐fluorocholine PET/CT was useful in guiding the surgical approach, enabling the surgeon to successfully locate and remove the ectopic parathyroid.

Primary hyperparathyroidism (pHPT) is a disorder of the parathyroid glands in which one or more of the glands secrete an excess amount of parathyroid hormone. pHPT is a common clinical problem usually first suspected because of the incidental finding of an elevated serum calcium concentration on biochemical screening tests. In addition, pHPT may be suspected in a patient with nephrolithiasis. The diagnosis is made by finding a frankly elevated parathyroid hormone (PTH) concentration in a patient with hypercalcemia.

Parathyroidectomy is indicated for all symptomatic patients, should be considered for most asymptomatic patients, and is more cost‐effective than observation or pharmacologic therapy.

Parathyroidectomy has a success rate of more than 95% for cure of primary hyperparathyroidism. In one series of 102 patients with persistent or recurrent hyperparathyroidism who required reoperation, over 10 years, reasons for failed parathyroid operations include tumor in ectopic position (53%), incomplete resection of multiple abnormal glands (37%), adenoma in normal position missed during previous surgery (7%), and regrowth of previously resected tumor (3%).^1^

Ectopic adenomas account for 3%‐4% of all parathyroid adenomas in clinical practice.^2^ They occur because parathyroid tissue may colocate with tissues that have a similar embryologic origin at the basis of the tongue and fail to fully migrate during development.

Ectopic parathyroid tissue remains a significant hurdle in the surgical management of hyperparathyroidism. In diagnostically challenging cases of persistent hyperparathyroidism, unusual ectopic locations of parathyroid adenomas must be considered in order to avoid numerous reoperative explorations and the associated risks to the patient.^3^

Accurate preoperative imaging can aid in detecting these ectopically located glands and allow a focused surgical approach with an even higher success rate.^4^ Presently, the main imaging techniques used for preoperative localization of a parathyroid adenoma are echography and ^99m^Tc‐sestamibi planar and/or single photon emission computed tomoscintigraphy (SPECT). Magnetic resonance imaging (MRI) scanning has also been used with success by some teams.^5^

Positron emission tomography/computed tomography (PET/CT) with ^18^F choline tracers now also emerges as a PET alternative to conventional ^99m^Tc sestamibi scintigraphy, with a better sensitivity because of the higher resolution of PET.^6^

We report a case of woman with persistent hyperparathyroidism after surgery, in whom several imaging techniques allowed to localize an adenoma in a very unusual retropharyngeal location.

## 2 CASE REPORT

A 30‐year‐old female was referred to the endocrinology department for symptomatic hypercalcemia. The patient had nausea, fatigue, and constipation since several months. There was no history of calcium disorders, kidney stones, fractures, osteoporosis, or ingestion of any drug that could be responsible for her hypercalcemia. Her family history was not contributory. Physical examination, including the neck, was unremarkable. Routine laboratory investigations showed a severe hypercalcemia (corrected serum calcium: 3.4 mmol/L, normal values (N):2.15‐2.5) with an intact parathyroid hormone serum concentration of 28.5pmol/l (third‐generation assay, N < 5.2). The serum phosphate was low (0.63 mmol/L, N:0.75‐1.39) and urinary calcium was 5.1 mmol/24 hours (N). The patient was deficient in vitamin D, with a 25‐hydroxyvitamin D at 30.7 nmol/L (recommended values > 50 nmol/L). A neck ultrasound failed to detect an enlarged parathyroid. A ^99m^Tc sestamibi scintigraphy showed a focus of radioactivity compatible with a hyperfunctioning lower right parathyroid. The pregnancy screening was done before any radiologic testing.

Thus, the lower right parathyroid gland was excised, with intraoperative testing for parathyroid hormone. Because the PTH value remained high the surgeon decided to remove 2.5 of the 3 remaining parathyroid glands, but the PTH value did not decrease. Pathology revealed 2 hyperplastic (the upper right gland with 1.3/0.6/0.3 cm and the upper left gland with 1.1/0.5/0.4 cm), 1 normal parathyroid gland (the right lower ‐ 1/0.7/0.3 cm), and thymus gland instead of left lower parathyroid.

**Figure 1:**
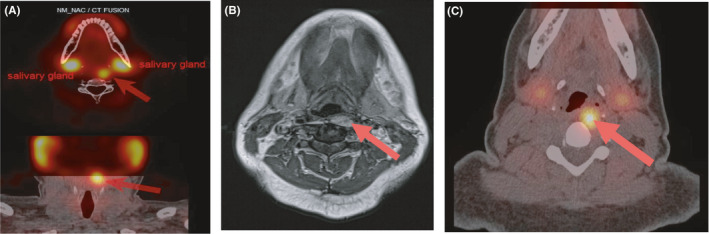
Images show a left retropharyngeal nodule, corresponds to a possible ectopic parathyroid lesion at: (A) ^99m^Tc sestamibi SPECT/CT, (B) MRI (T1 axial), and (C) 18F‐fluorocholine PET/CT respectively

**Figure 2:**
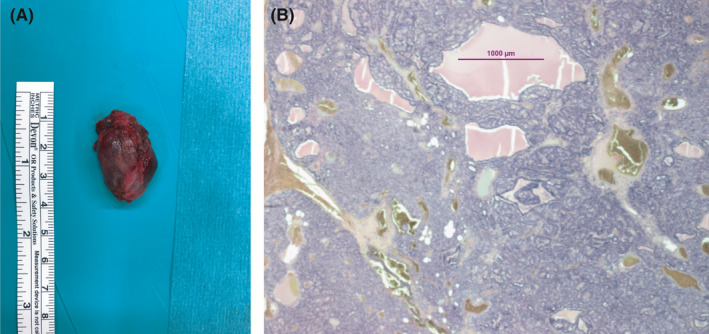
Anatomo‐pathological aspect of the ectopic parathyroid adenoma: (A) macroscopic aspect: an encapsulated lesion (3.2 × 1.7 × 1.2 cm, weighty 3.9 g); (B) microscopic aspect: an encapsulated lesion composed of uniform cells arranged in the form of solid nests and pseudo follicles

Postoperatively, serum calcium and PTH remained increased. A ^99m^Tc sestamibi scan was repeated but did not show an ectopic mediastinal parathyroid adenoma. However, there was a concentration of sestamibi at the upper limit of the field, corresponding to a pharyngeal bump on the CT. A mass was also detected at MRI in this position, anterolateral to the left part of C4 measuring 18 × 10 × 35 mm. This nodule was a T1‐hypointense and T2‐hyperintense lesion. To confirm this very unusual location, the patient underwent a ^18^F choline PET/CT, as a part of research protocol, which showed a left retropharyngeal hyperactivity at the level of C4, corresponding on the CT to a mass which was about 12 × 20 mm in diameter deforming the posterior wall of the hypopharynx opposite to the left piriform sinus (Figure 1). The patient underwent a successful removal of the lesion by transpharyngeal approach after intravenous administration of ^99m^Tc sestamibi 6 hours before surgery. A handheld gamma‐probe was used by the surgeon to exactly localize the lesion. After the placement of an orthostatic mouth opener and localization of the lesion using the sentinel node locator, the surgeon did the incision of the left posterior pharyngeal mucosa in front of the epiglottis, the dissection of the prevertebral muscles and spotting of a large lesion. Pathology confirmed the presence of a parathyroid adenoma (3.2 × 1.7 × 1.2 cm, weighting 3.9 g) (Figure 2). As expected, the patient became hypocalcemic postoperatively (serum calcium at 1.99mmol/L with intact parathyroid hormone <0.5 pmol/L and normal phosphorous (0.86 mmol/L)), requiring treatment with calcium and active vitamin D. Eight month after the second operation and the removal of the ectopic parathyroid, the patient has a normal PTH and calcium without treatment.

The genetic tests showed no mutations in the CDKN1B, MEN, and RET genes.

## 3 DISCUSSION

Ectopic adenomas have been reported to account for 4%‐16% of patients with hyperparathyroidism and are the cause of a significant proportion of failed primary surgery for pHPT.^3^

The parathyroid glands originate from epithelial cells of the pharyngeal pouches. The superior parathyroid glands develop from the fourth pharyngeal pouch, which is also responsible for producing the thymus gland whereas the inferior parathyroid glands arise from the third pharyngeal pouch, which also produces the thyroid gland. During normal development, the parathyroid glands separate and travel in a caudal direction with the thymus, which migrates further into the mediastinum. Ectopic parathyroid gland can occur anywhere along this migration path.^3^, ^7^ In the quite rare situation when the lower parathyroid fails to migrate normally, they can reside very high in the neck like in the case of our patient.

The ectopic gland may be one of the four parathyroid glands, like in our case, or it may be a supernumerary gland. In one series of 102 patients with persistent or recurrent hyperparathyroidism, ectopic glands were found in the paraoesophageal position (28%), in the mediastinum (26%), intrathymic (24%), intrathyroidal (11%), in the carotid sheath (9%), and in a high cervical position (2%).^1^

Parathyromatosis is a rare cause of persistent or recurrent hyperparathyroidism. It is characterized by small nodules and nests of hyperfunctioning parathyroid tissue in soft tissue caused by seeding or implantation of parathyroid tissue during surgical removal or by overgrowth of embryologic nests of parathyroid tissue left behind in development.

Thus, our patient had an ectopic parathyroid adenoma in a very unusual location, and this case highlights the pivotal role of medical imaging after surgery has failed to cure HPT. In particular, it shows that, though the most frequent location of ectopic PT is the mediastinum, imaging must cover the complete migration path of the embryonic gland, from the pharynx to the xyphoid appendix. Similar cases were presented by Batchala et al^8^ and Gallagher et al^9^ who highlight the retropharyngeal space as an important ectopic site of the ectopic parathyroid, limitation of ultrasonography, and Tc‐99m‐sestamibi planar scintigraphy in identifying retropharyngeal parathyroid adenomas.

Reported accuracy of preoperative localization imaging for pHPT varies. In the meta‐analyses of Cheung et al, ultrasound had pooled sensitivity and positive predictive value (PPV) of 76.1% (95% CI 70.4%‐81.4%) and 93.2% (90.7%‐95.3%), respectively. Single photon emission computed tomography (SPECT) with ^99m^Tc sestamibi had pooled sensitivity and PPV of 78.9% (64%‐90.6%) and 90.7% (83.5%‐96.0%), respectively. Accuracy may be improved by 4D‐CT with a sensitivity and PPV of 89.4% and 93.5%, respectively.^5^ ^99m^Tc sestamibi scan is negative in 12%‐25% of patients with HPT. Sestamibi scanning is often unrevealing, or misleading, in patients with parathyroid hyperplasia, multiple parathyroid adenomas or ectopic adenoma.

Four‐dimensional computed tomography (4D‐CT) scans take advantage of the rapid contrast uptake and washout that is characteristic of parathyroid adenomas for precise anatomic localization. The primary disadvantage of 4D‐CT is the radiation exposure, which, compared with sestamibi imaging, results in a >50‐fold higher dose of radiation absorbed by the thyroid. 4D‐CT is particularly useful in the reoperative setting when initial imaging with sestamibi is negative. Several studies report an added value of 18F‐fluorocholine (FCH) PET/CT in the localization of parathyroid adenoma.^10^, ^11^, ^12^, ^13^ The PET 18F‐choline has not yet entered in the routine imaging study for primary hyperparathyroidism but it is performed in research protocols. A growing body of literature has examined the role of radiolabelled choline PET, which can be combined with CT (PET/CT) or magnetic resonance imaging (PET/MRI). As a phospholipid analogue, radiolabelled choline is integrated into newly synthesized membranes of proliferating cells, and its uptake is increased by upregulation of choline kinase. Upregulation of phospholipid‐dependent choline kinase has been shown to be related to PTH secretion in HP. The increase in phosphatidylcholine turnover in HP is the rationale for using radiolabelled choline PET for HP detection in patients with HPT.^14^ Because of the better resolution of PET imaging compared to SPECT, 18F‐FCH, which concentrates in hyperfunctionning parathyroid tissue, is a promising upcoming tracer for the detection of parathyroid adenomas, especially when multiple or small. With reported sensitivity, PPV and accuracy of FCH PET/CT were 94.1%, 97.9%, and 92.4%, respectively,^15^ FCH PET/CT has the potential to become a standard investigation in the detection of parathyroid lesions ^11^ and should be recommended for patients with negative or inconclusive results on conventional imaging.^16^ Bilateral cervical exploration could be avoided in the majority of patients.^17^ Prospective studies are still needed to specify its role in first‐line settings along with cost‐effectiveness studies.^13^, ^18^ Large multicentre studies and cost‐effectiveness analyses are needed to better define the role of this imaging method in this setting.^14^, ^16^

The patient was operated using a transpharyngeal approach. After administration of 99mTc sestamibi, radioguided surgery with a gamma‐probe allowed a precise location of the ectopic adenoma. This transpharyngeal approach is rarely used because the dissection is done blindly by traction of its upper or lower pole, without control of the internal carotid and with a risk of rupture of the capsule with incomplete excision or dissemination. However, in this case, it allowed to remove rapidly a parathyroid adenoma and avoided to return to a site which had been operated on previously. At this time, the patient is cured.

In conclusion, we reported a case with persistent hyperparathyroidism after cervical surgery, due to an ectopic retropharyngeal parathyroid adenoma for which different imaging techniques and particularly 18F‐fluorocholine PET/CT were essential in guiding the surgical approach and enabling the surgeon to successfully locate and remove the ectopic parathyroid.

## CONFLICT OF INTEREST

All authors state that they have no conflicts of interest.

## AUTHOR CONTRIBUTIONS

LI: wrote the first draft of the manuscript. LI, RK, RD, LS, PB, ASH, AD, MH, and CM: revised subsequent versions of the manuscript. All authors read and approved the final version of the paper. LI accepts responsibility for the integrity of the data analyses.

## ETHICAL APPROVAL

The consent has been obtained from patient after full explanation of the purpose and nature of all procedures used.

## Data Availability

The datasets used and/or analyzed during the current study are available from the corresponding author on reasonable request.
